# Panoramic Near-Infrared Imaging Device for Peripheral Vascular Mapping

**DOI:** 10.3390/s26154895

**Published:** 2026-08-03

**Authors:** Bendegúz Juhos, Imre Vida, Csaba Csobay-Novák

**Affiliations:** 1 Department of Interventional Radiology, Heart and Vascular Centre, Semmelweis University, 1122 Budapest, Hungary; 2 Department of Electronics, Information and Bioengineering, Politecnico di Milano, 20133 Milano, Italy; imre.vida@mail.polimi.it

**Keywords:** near-infrared imaging, vein detection, vascular mapping, non-invasive diagnostics, superficial veins

## Abstract

Longitudinal mapping of the superficial peripheral vascular network is clinically relevant for conditions such as venous insufficiency, superficial venous thrombosis, and localized hemorrhagic injury; yet existing modalities are either operator-dependent, costly, or unable to provide wide-field coverage. We designed and demonstrated the feasibility of a relatively quick, cost-effective, automated panoramic near-infrared (NIR) imaging system operating at 850 nm, with a total acquisition time of 30 s. The prototype integrates a motorized rotational acquisition frame, co-localized illumination, lateral mirror enhancement, and a time-of-flight ranging sensor. A custom image processing pipeline combining contrast-limited adaptive histogram equalization (CLAHE), black-hat morphological filtering, Otsu binarization, structure-tensor-based diameter quantification, and photogrammetric 3D reconstruction was applied to data from five healthy volunteers. Optimal acquisition parameters (30 ms exposure, analog gain 3.5) were identified via systematic characterization. The pipeline reliably isolated venous structures of the 35 cm field of view, yielding a mean superficial vein diameter of 2.69 ± 1.48 mm, consistent with published anatomical references. A panoramic texture-mapped 3D surface reconstruction of the limb was generated for each participant. The system provides a rapid, operator-independent panoramic view of superficial veins suitable for future longitudinal monitoring, offering a compelling complementary tool to duplex ultrasound for tracking morphological and volumetric vascular changes.

## 1. Introduction

The early detection of morphological and functional abnormalities in the superficial vascular network in conditions such as superficial venous thrombosis, venous insufficiency, or superficial hemorrhagic injury is essential for timely and appropriate therapeutic decisions. Although duplex ultrasound remains the gold-standard diagnostic procedure [1], providing limited field of view cross-sectional imaging and flow measurements, its high cost and the need for a specially trained operator limit its widespread longitudinal application. Recently, alternative low-cost modalities, such as microwave-based contact sensors, have also been investigated for localized vein detection [2], but are similarly challenging to extend to larger areas. Other imaging approaches, such as catheter angiography, computed tomography angiography, magnetic resonance angiography, optical coherence and photoacoustic tomography, may provide information about the whole vascular network; however, these techniques are often slow and require contrast-agent administration [3], radiation exposure, or even more expensive specialized machinery. For the assessment of superficial and subcutaneous vascular structures, Near-Infrared (NIR) Imaging has emerged as a promising alternative [4,5,6,7]. The cost-effective, rapid, non-invasive wide-field imaging capabilities make it particularly attractive for initial vascular assessment and longitudinal patient monitoring.

**Figure 1:**
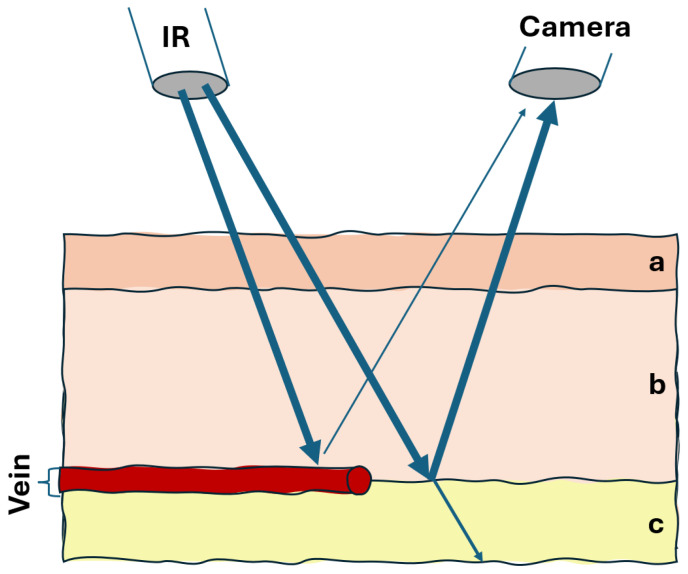
Simplified skin cross section with different layers ((**a**): Epidermis, (**b**): Dermis, (**c**): Hypodermis). The vein absorbs more infrared (IR) light and reflects less into the camera (as illustrated by the arrows), making the vascular mapping possible.

The human skin is an optically complex tissue [8] characterized by multiple scattering layers, heterogeneous refractive indices, and a diverse population of chromophores, primarily hemoglobin and melanin [9]. In the wavelength range between 740 nm and 940 nm, venous blood (deoxygenated hemoglobin) absorbs significantly more light than the surrounding dermal and subdermal tissues (where water and melanin absorption is lower), causing veins to appear as dark structures on the camera image [10,11,12]. See Figure 1.

According to the literature, depending on the wavelength and tissue density, NIR light can penetrate to depths of 2.6–15 mm [4], making it ideal for examining the superficial veins of the hands and feet, which are typically situated 2–4.1 mm deep [13].

Devices operating in the NIR range have become widely adopted in everyday clinical practice, mainly for photoplethysmography-based pulse oximetry and as assistive tools for venipuncture. Beyond these standard applications, the optical contrast generated by tissue light scattering and localized absorption also creates an excellent opportunity for the non-invasive optical mapping of venous pathologies, including occlusions, inflammations, and hemorrhagic injuries. Despite this broader clinical potential, recent literature on vascular optical devices has largely concentrated on highly specific or localized tasks. Significant academic efforts have focused on providing real-time guidance and automation for existing medical procedures, such as venipuncture and contrast agent administration [14,15,16], or on monitoring short-term dynamic physiological changes during vascular occlusion [17].

Parallel to these clinical developments, this technology has mostly been exploited outside the medical field. A substantial body of research has focused on the development of automated NIR vein-imaging systems designed specifically for biometric identification and security applications [7,18,19,20]. Unlike medical diagnostic tools, these systems prioritize pattern matching and liveness detection rather than the assessment of tissue pathology.

Alternatively, other studies have pursued highly detailed vascular assessment using advanced, multi-wavelength hyperspectral imaging systems [21,22,23,24,25]. Although these high-end modalities offer exceptionally detailed spatial and spectral data, they often entail significant complexity, computational overhead, and high costs, limiting their routine application.

Our aim is to develop and evaluate an automated imaging system that can be easily deployed in routine clinical settings. By overcoming the narrow field-of-view, operator dependency, and high costs of current devices, we aim to supplement currently available diagnostic techniques with a rapid, objective, first-line assessment tool for longitudinal vascular tracking.

- To create a panoramic map of the superficial vascular network
- Provide a qualitative 3D topographical visualization of the imaged limb.
- Develop tools to enable easy characterization of vessels, for potential diagnostic value.

## 2. Materials and Methods

### 2.1. Data

As a baseline validation of the system’s spatial resolution and measurement accuracy, we first tested the optical pipeline against known targets utilizing paper-printed fractal trees described below.

This study was conducted at a single tertiary vascular center. Ethical review and approval were waived by departmental authority, and all procedures were performed in accordance with the Declaration of Helsinki. Written informed consent was obtained from all 5 volunteers prior to their participation.

Fully non-invasive and non-contact imaging was performed using the experimental instrument (described in Section 2.2) with participants in a resting state while seated in close proximity to the system. During this interval, the device acquired several images from multiple angles using infrared illumination, which were subsequently stored for offline analysis. The total imaging duration was approximately 30 s per participant. Standard vital sign measurements, including blood pressure (Omron M3 Intellisense, Omron Healthcare Co., Ltd., Kyoto, Japan) and oxygen saturation (SpO2) using a Hartmann Veroval pulse oximeter (Paul Hartmann AG, Heidenheim, Germany), were recorded immediately preceding or following the imaging procedure (as shown in Table 1) to ensure temporal proximity between the hemodynamic data and the imaging acquisition. These parameters were obtained to confirm a healthy, uniform baseline condition.

### 2.2. Device

**Figure 2:**
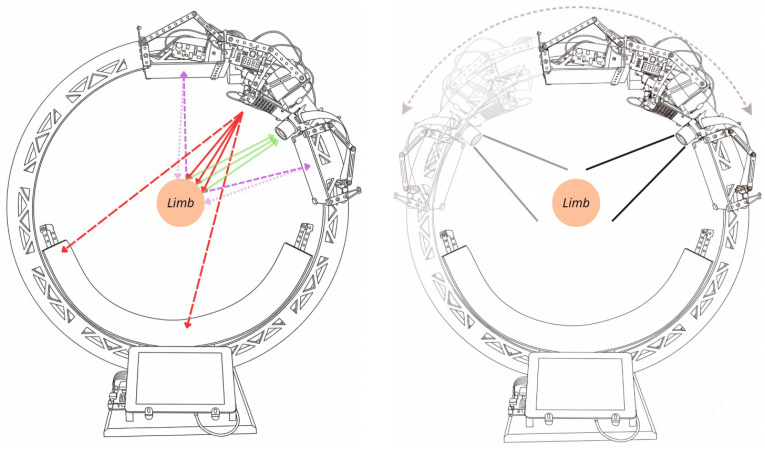
CAD schematic of the panoramic acquisition mechanism. (**Left**): front view showing the co-localized illumination (850 nm LED) and NoIR camera assembly, with the limb centered in the imaging plane; colored arrows indicate the illumination path (red), reflected near-infrared (NIR) signal (green), and lateral mirror-redirected backscatter (purple dashed). (**Right**): front view illustrating the motorized rotation arc and the field of view (FOV) of the camera module.

**Figure 3:**
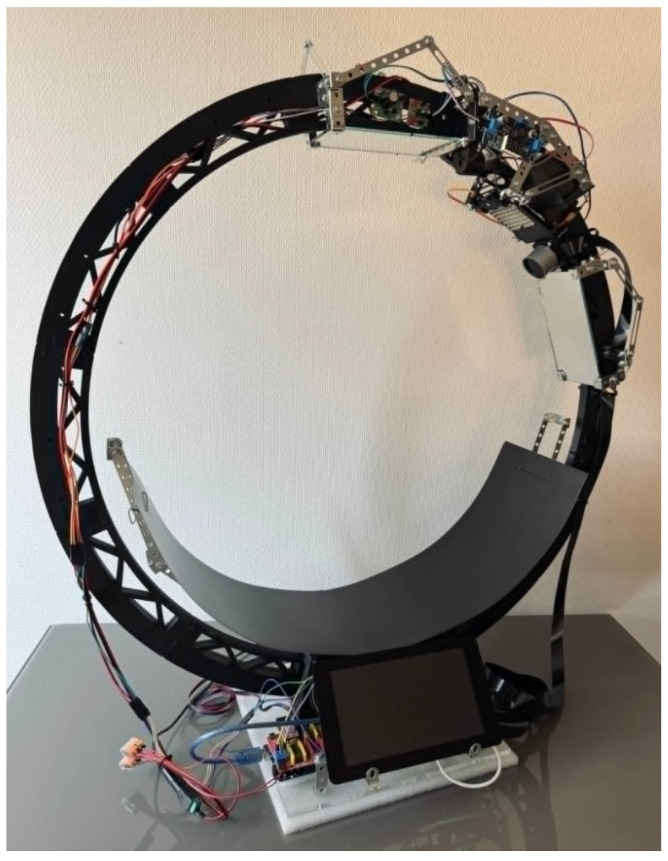
Prototype of the proposed system.

**Figure 4:**
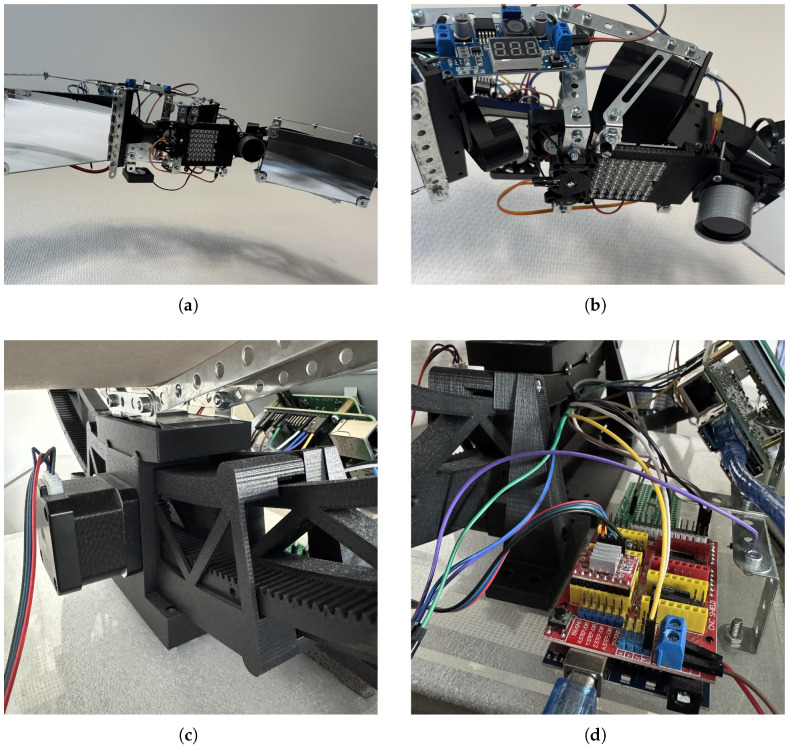
Photographs of key hardware subassemblies. (**a**) Lateral mirror pair mounted adjacent to the camera aperture for photon-collection enhancement. (**b**) Camera unit: Raspberry Pi Camera Module 3 NoIR (Sony IMX708) mounted in its 3D-printed housing with the 850 nm LED array. (**c**) Stepper motor (NEMA17) and 3D-printed gear train responsible for the rotational panoramic sweep. (**d**) Motor control electronics: Arduino-based A4988 driver board and MOSFET switching logic for synchronized illumination triggering.

The prototype utilizes a reflective imaging architecture with co-localized detection and illumination as shown in Figure 2. The imaging sensor is a calibrated Raspberry Pi Camera Module 3 NoIR (Sony IMX708, 12 MP) (Cambridge, UK). A precise sensor-to-subject distance was measured with a VL53L0X ranging sensor. Illumination is provided by a 1 W, 850 nm LED source. To maximize photon collection, lateral mirrors are positioned near the optics, oriented to redirect backscattered photons toward the target, thereby enhancing the signal-to-noise ratio (see Appendix A). The motorized base rotates the main frame with the sensor assembly to perform the acquisition, which is initiated and managed through a 7-inch integrated touchscreen and the internal computer (Raspberry Pi 4B). The prototype of the system can be seen on Figure 3 and Figure 4. The system ensures precise synchronization between mechanical actuation and image acquisition. The distance between the sensors and the target is recorded for each frame to account for distance-dependent signal variations during the panoramic sweep, and the illumination is only active for the short duration of the image capture window.

To map the spatial resolution of the optical imaging system as a function of depth, a geometric model of the camera’s field of view (FOV) was established. The physical dimension represented by a single pixel varies linearly with the working distance from the lens vertex. For a system characterized by an angular horizontal FOV (θ) and a horizontal pixel resolution (*N*), the spatial footprint of an individual pixel ($S_{f}$) at a target distance (*d*) was determined using the following geometric relationship:

(1) $$S_{f}=\frac{2\cdot d\cdot \tan\left(\frac{\theta }{2}\right)}{N}$$

where $S_{f}$ represents the physical width covered per pixel, *d* denotes the perpendicular distance from the camera lens to the object plane, which was measured for each frame using the mounted VL53L0X ranging sensor, θ is the horizontal FOV ($75^{\circ }$), and *N* is the horizontal pixel sensor resolution. The measured distance data is crucial for converting pixel-based measurements into real-world units and ensuring accurate spatial reconstruction.

**Figure 5:**
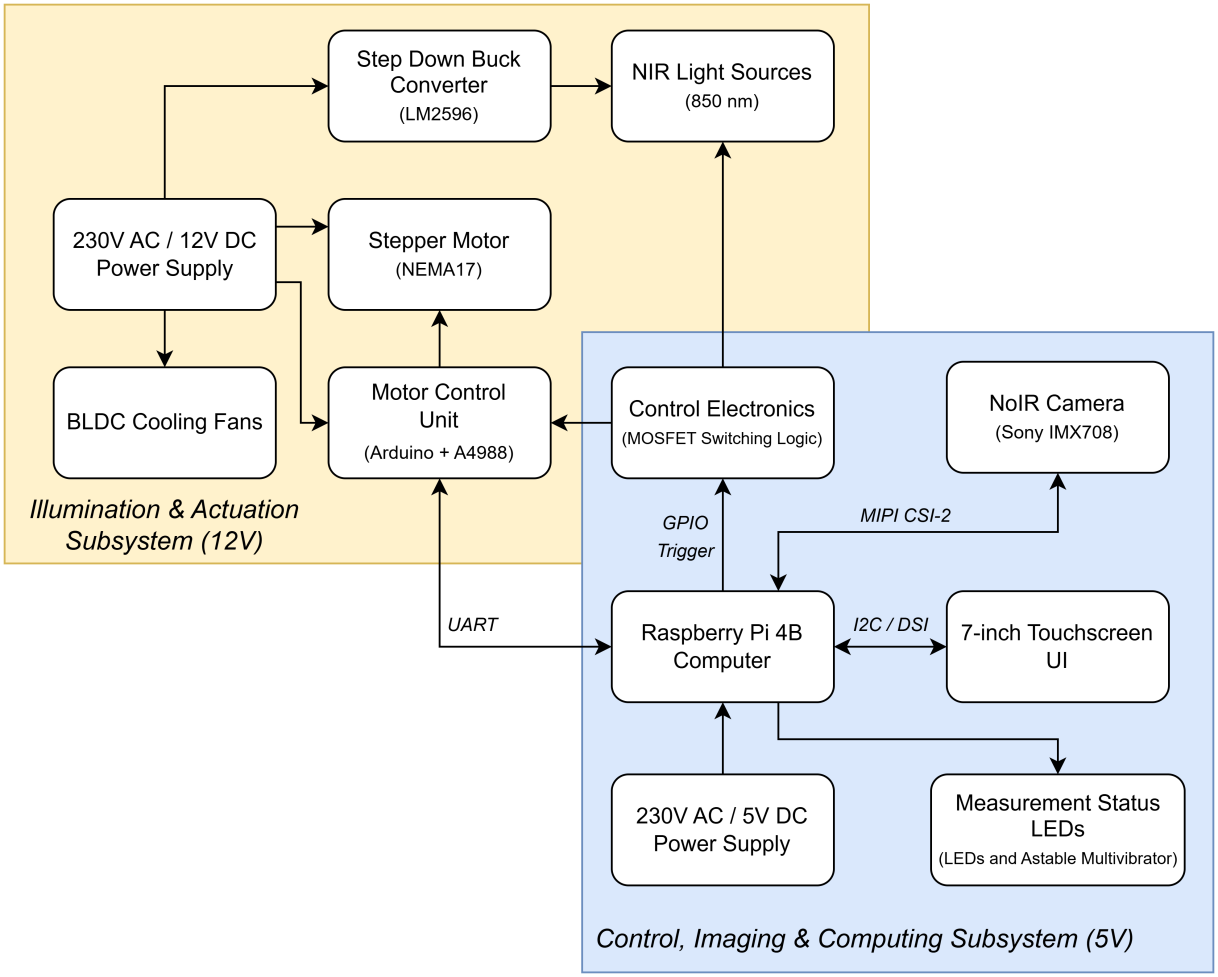
Schematic block diagram of the imaging system hardware architecture, illustrating the separation of the 12 V Illumination and Actuation Subsystem from the 5 V Control, Imaging and Computing Subsystem. Arrows indicate the flow of control signals (UART, GPIO) and data (MIPI CSI-2, I2C/DSI) between the system components.

The schematic diagram of the primary hardware components is presented in Figure 5. The system architecture is divided into two distinct power domains: an Illumination and Actuation subsystem (12 V) and a Control and Computing subsystem (5 V). The former manages the mechanical movement, the internal cooling and the illumination, separated from the latter, that is built around the onboard computer responsible for the user interface and the synchronization of the data acquisition.

To prevent distortion caused by dynamic autofocus, the lens was locked at a fixed focal length (near-infinity). Automatic white balance was disabled and the exposure was set to 30 ms with an analog gain of 3.5. These parameters were determined by characterizing the device and selecting optimal parameters for the desired exposure time, balancing contrast, signal-to-noise ratio (SNR), and mitigation of motion blur. In the context of this study, the extracted vascular network is treated as the foreground signal and the surrounding skin tissue and background as the noise. Consequently, the SNR is conceptualized to be equivalent to the signal-to-background ratio (detailed in Appendix A).

### 2.3. Processing

The image processing pipeline was designed to enhance vascular contrast and normalize the raw data acquired during the panoramic sweep. All processing was done in Python 3.12 using the OpenCV library. Images were converted to grayscale and uniformly scaled. The limb silhouette was extracted using dynamic brightness-thresholding (with an empirically selected threshold of 35% of maximum intensity) to create a Region Of Interest (ROI). The background was masked, and a 15 px size morphological dilation was applied to ensure complete coverage of the limb.

This information has been used with the imaging angle information to obtain the pointwise diameter of the limb from different angles. To estimate the volume of the imaged limb, standard photogrammetry-based 3D reconstruction was performed on this sequence. This results in a solid homogeneous object; consequently, its utility is limited to surface circumference and total volume measurements, rather than a comprehensive cross-sectional reconstruction.

Vascular visibility was optimized via a dual-stage Contrast Limited Adaptive Histogram Equalization (CLAHE) [26] (initial: clipLimit = 4.0, 10 × 10 grid; secondary: clipLimit = 1.5, 8 × 8 grid), which is frequently used in recent literature [16,27]. A 3 × 3 median filter was applied to suppress surface hair artifacts and sub-pixel-sized vascular structures that cannot be properly resolved, while preserving macroscopic vascular structures.

A black-hat morphological filter was used to isolate dark vascular structures. The black-hat operation calculates the difference between the morphological closing of the image and the original image. For a grayscale input image *I*, this is computed using local maximum and minimum pixel values within a 17×17 spatial neighborhood window $W_{17}$:

(2) $$I_{\mathrm{bh}}\left(x,y\right)=\min_{\left(i,j\right)\in W_{17}}\left[\max_{\left(m,n\right)\in W_{17}}I\left(x+i+m,y+j+n\right)\right]-I\left(x,y\right),$$

the variables (m,n) and (i,j) represent the discrete spatial offsets within the structuring element $W_{17}$ for the inner morphological dilation and outer morphological erosion, respectively. This was followed by a +50% linear contrast amplification, which scaled the pixel intensities at each coordinate pair (x,y) according to the relation:

(3) $$I_{\mathrm{amp}}\left(x,y\right)=1.5\cdot I_{\mathrm{bh}}\left(x,y\right),$$

Binarization has been performed using Otsu’s method [28]. Briefly: to find the optimal threshold $t^{*}$, the normalized probability $p_{i}$ of each gray level *i* (ranging from 0 to L−1, where L=256) is defined using the pixel counts $n_{i}$ and total pixels *N* as $p_{i}=n_{i}/N$. The cumulative sums for the background probability $\omega_{0}\left(t\right)$ and foreground probability $\omega_{1}\left(t\right)$ are expressed as:

(4) $$\omega_{0}\left(t\right)=\sum_{i=0}^{t}p_{i},\;\omega_{1}\left(t\right)=\sum_{i=t+1}^{L-1}p_{i},$$

while the respective mean intensities $\mu_{0}\left(t\right)$ and $\mu_{1}\left(t\right)$ for both classes are defined by the weighted sums:

(5) $$\mu_{0}\left(t\right)=\frac{1}{\omega_{0}\left(t\right)}\sum_{i=0}^{t}i\cdot p_{i},\;\mu_{1}\left(t\right)=\frac{1}{\omega_{1}\left(t\right)}\sum_{i=t+1}^{L-1}i\cdot p_{i}.$$

The optimal binarization threshold $t^{*}$ is selected by maximizing the between-class variance:

(6) $$t^{*}=\arg\max_{t}\left\{\omega_{0}\left(t\right)\omega_{1}\left(t\right){\left[\mu_{0}\left(t\right)-\mu_{1}\left(t\right)\right]}^{2}\right\},$$

which maps $I_{\mathrm{amp}}\left(x,y\right)$ to a binary mask $M_{\mathrm{bin}}\left(x,y\right)$ such that $M_{\mathrm{bin}}\left(x,y\right)=1$ if $I_{\mathrm{amp}}\left(x,y\right)\geq t^{*}$, and 0 otherwise. Fine-tuned morphological opening (5 × 5 kernel) removed residual noise. This opening operation suppresses small background noise structures by sequentially applying a minimum filter followed by a maximum filter over a 5×5 window $W_{5}$:

(7) $$M_{\mathrm{final}}\left(x,y\right)=\max_{\left(m,n\right)\in W_{5}}\left[\min_{\left(i,j\right)\in W_{5}}M_{\mathrm{bin}}\left(x+m+i,y+n+j\right)\right].$$

Similarly, (i,j) and (m,n) denote the spatial offsets within the structuring element $W_{5}$ for the inner erosion and outer dilation steps. The sequential frames were stitched into a single panoramic projection. Furthermore, these frames served as the texture map for the 3D reconstruction of the limb. To facilitate topological analysis of the vascular network, a thinning algorithm was applied to the segmented images, generating a single-pixel-wide midline skeleton of the venous system. This skeletonization provides a representation of the vascular morphology, enabling the objective evaluation of branching patterns and junctions.

Vein cross-sectional diameters were quantified using an automated Python pipeline applied to CLAHE-enhanced grayscale imagery and corresponding binary skeleton maps. Initial spatial coordinates were extracted from all foreground pixels of the skeletonized images. To determine the local vascular geometry at each coordinate, a structure tensor, *S*, was computed from Sobel-derived spatial gradients ($G_{x}$ and $G_{y}$) within a localized 17×17 pixel window (radius = 8 pixels):

(8) $$S=\left[\begin{array}{cc} \sum G_{x}^{2} & \sum G_{x}G_{y}\\ \sum G_{x}G_{y} & \sum G_{y}^{2} \end{array}\right].$$

Eigenvalue decomposition of the structure tensor yielded the local orthogonal (cross-sectional) and parallel (longitudinal) vectors of the vein. Specifically, the eigenvector $v_{⊥}$ corresponding to the largest eigenvalue $\lambda_{1}$ (representing the direction of highest gradient variance) dictated the orthogonal direction:

(9) $$Sv_{⊥}=\lambda_{1}v_{⊥}.$$

Along this orthogonal vector, a 41-pixel 1D intensity profile was extracted at sub-pixel resolution using bilinear spatial interpolation. The profile was subjected to a 5-point moving average filter, after which the algorithm found the local intensity minimum representing the darkest center of the vessel. Vessel boundaries were identified by calculating the first spatial derivative of the 1D profile to locate the points of maximum gradient (steepest ascent and descent) adjacent to the center minimum.

To account for potential morphological asymmetry and noise, the local width, *w*, was calculated as twice the distance from the central minimum to the physically closest boundary. To further ensure measurement stability, this calculation was performed at the central coordinate $p_{0}$ and its two immediate adjacent pixels ($p_{-1}$ and $p_{+1}$) along the longitudinal vector. The final theoretical diameter, *D*, was defined as the average across these three contiguous points, converted into millimeters using $S_{f}$ from Equation (1):

(10) $$D=S_{f}\cdot \frac{1}{3}\sum_{i=-1}^{1}w\left(p_{i}\right).$$

**Figure 6:**
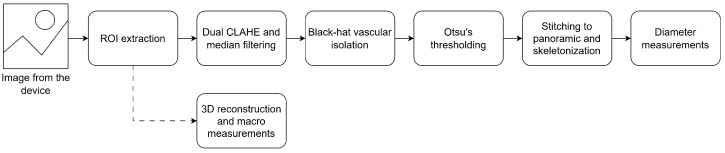
Simplified flowchart of the automated image processing pipeline.

The simplified summary of the above-described processing pipeline is illustrated in Figure 6.

To validate the accuracy of the automated diameter quantification pipeline on known size targets, synthetic vascular phantoms were generated as asymmetric fractal trees. A Python script utilizing a recursive algorithm was developed to simulate the branching morphology and caliber variations of superficial venous networks. Starting from a root segment (3, 4 or 5 mm) with an initial width and a length of 45.0 mm, the algorithm recursively generated bifurcating branches up to a maximum depth of 8 or 10 iterations. At each junction, the daughter segments were deflected by different angles relative to the parent branch, while their lengths and widths were scaled down by constant decay multipliers (0.67 and 0.68, respectively). Four distinct fractal patterns with mathematically predefined morphological properties were synthesized.

**Figure 7:**
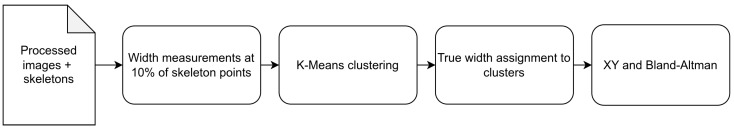
Simplified flowchart of the validation procedure using synthetic fractal phantoms.

After acquiring images of the synthetic phantoms (which were rolled up to 7 cm diameter tubes to represent generic limbs), the above-described pipeline was applied, and then random points were uniformly selected across the tree structure. The target sizes seen by the device were measured at these locations and compared to the known width values. Known values were assigned to each point using an initial K-Means clustering on the measurements followed by a manual ground truth assignment to the clusters (k was selected to be equivalent to the depth of the current fractal). The agreement was evaluated using an X-Y plot, and Bland-Altman analysis. This workflow is summarized in Figure 7.

After the processing pipeline, the quantified human data were projected onto an interactive graphical interface, where measurement segments were visualized using a diameter-scaled colormap. The interface enabled real-time automatic statistical evaluation via an active histogram. Additionally, it provides tools for quality control by allowing the user to interactively append new measurement coordinates or explicitly exclude irrelevant anatomical regions.

## 3. Results

**Figure 8:**
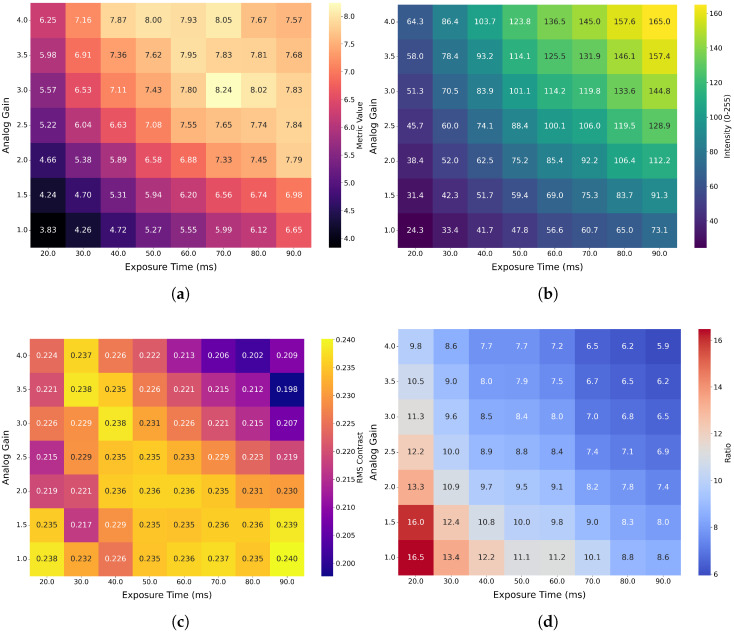
Device characterization heatmaps across varying exposure times and analog gains: (**a**) Mean black-hat response; (**b**) Region of Interest (ROI) mean intensity; (**c**) RMS contrast (Standard Deviation/Mean); and (**d**) Signal-to-Noise Ratio (SNR) of the ROI versus background. Values are calculated based on human data.

To determine the optimal hardware settings for clinical deployment, the imaging device was characterized across a matrix of exposure times (20–90 ms) and analog sensor gains (1.0–4.0). Figure 8 illustrates the resulting heatmaps for mean black-hat response, ROI intensity, Root Mean Squared (RMS) contrast, and SNR. Because the system relies on motorized rotational acquisition, exposure time had to be strictly minimized to prevent motion blur. Based on the characterization data, an exposure time of 30 ms paired with an analog gain of 3.5 was selected. The decision criteria for these parameters followed a two-step optimization. Firstly, we wanted to achieve at least 30 frames per second to keep the overall acquisition time low. Secondly, we empirically selected the gain to be 3.5 as a tradeoff between SNR and high vascular contrast, without over-amplifying background sensor noise. As demonstrated in Figure 8c,d, these parameters provided an optimal compromise, yielding high vascular contrast and a strong SNR while maintaining sufficient temporal resolution for the panoramic sweep. The current prototype requires roughly 30 s to finish the full panoramic sweep, including travel and imaging times. Every acquired image captures an approximately 35 cm segment of the limb defined by the FOV as discussed in Section 2.2.

**Figure 9:**
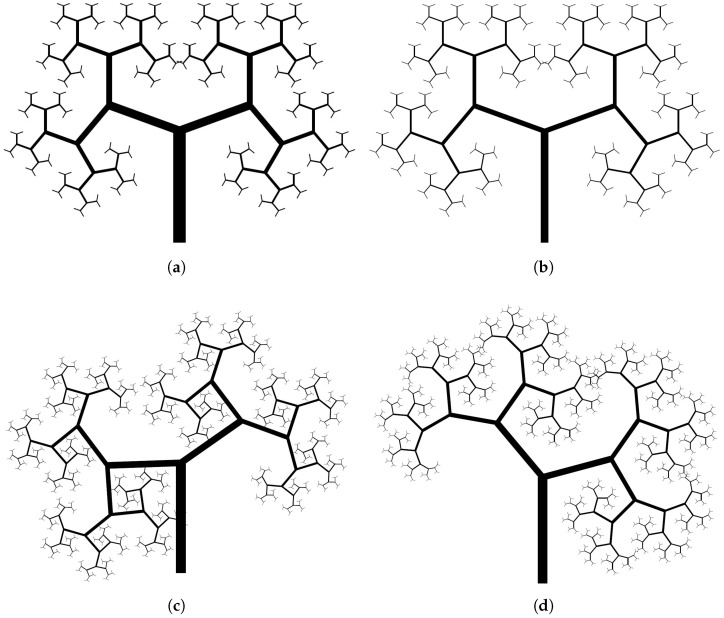
Fractal phantoms generated for the quantitative validation of the diameter measurement: (**a**) Fractal map with depth: 8, left-angle: $70^{\circ }$, right-angle: $-70^{\circ }$, start-width: 5 mm. (**b**) Fractal map with depth: 8, left-angle: $70^{\circ }$, right-angle: $-70^{\circ }$, start-width: 3 mm. (**c**) Fractal map with depth: 10, left-angle: $92^{\circ }$, right-angle: $-55^{\circ }$, start-width: 4 mm. (**d**) Fractal map with depth: 10, left-angle: $40^{\circ }$, right-angle: $-75^{\circ }$, start-width: 4 mm.

The synthetic fractal phantoms illustrated on Figure 9 were generated for the quantitative validation of the diameter measurement algorithm. The four distinct morphologies serve as an exact geometric ground truth to assess the accuracy of the structure-tensor-based pipeline.

**Figure 10:**
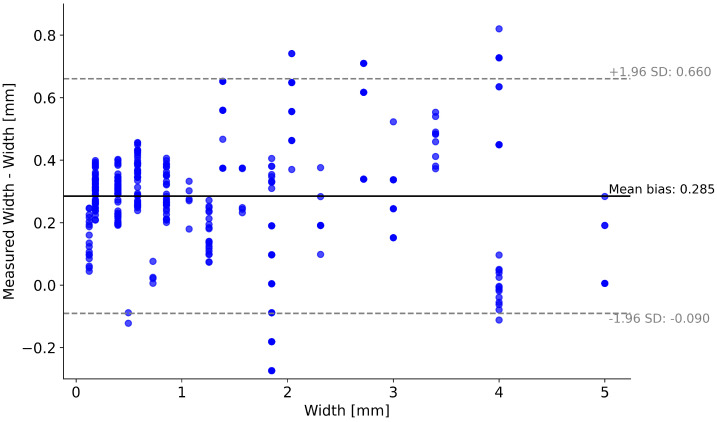
Bland−Altman plot comparing the measurements across all synthetic fractal trees against known values.

Due to the geometry of the samples, a higher number of distinct values were covered at smaller widths. The Bland-Altman plot shown in Figure 10 incorporated data from all synthetic fractal trees.

**Figure 11:**
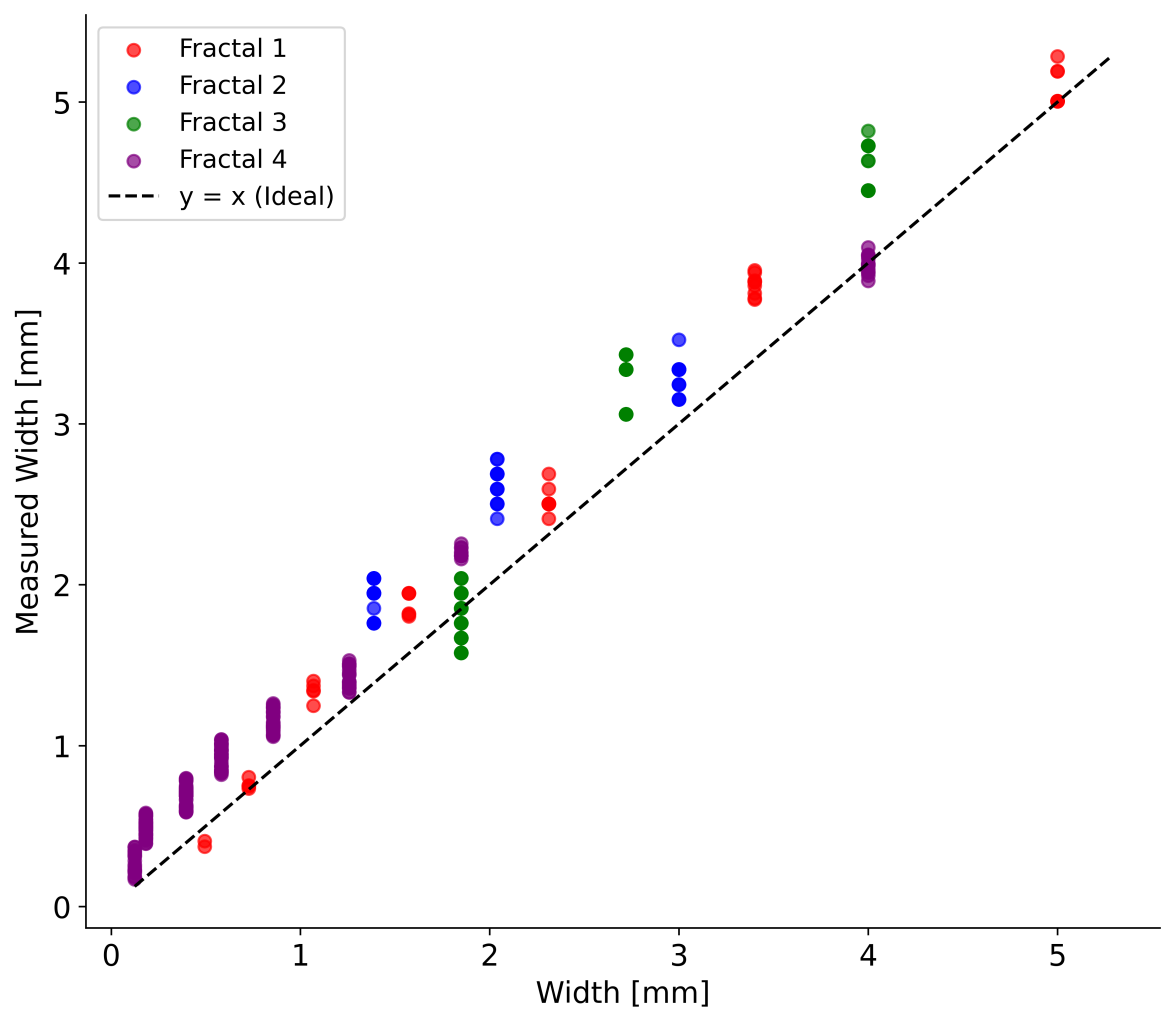
Correlation plot showing measured width against known values. Individual fractal phantoms are separated by colors.

Figure 11 presents a correlation plot comparing the measured width with the ground truth data, colored by data source. The data points are tightly clustered around the y = x line for each dataset indicating a strong positive correlation.

**Figure 12:**
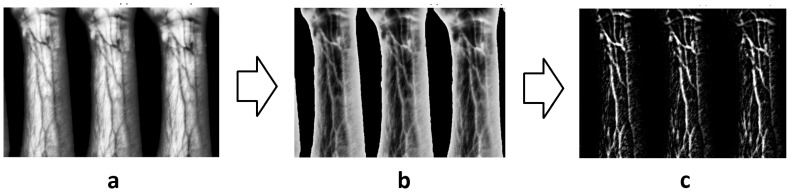
Main image processing steps to extract the vascular network. (**a**) Contrast-limited adaptive histogram equalization (CLAHE)-enhanced near-infrared (NIR) image of the limb. (**b**) Inverse CLAHE and median filtered image. (**c**) Extracted and binarized vascular network achieved via black-hat morphological filtering and Otsu’s thresholding. Example shown on a human subject.

The image processing pipeline applied to human data successfully mitigated uneven illumination and isolated the superficial venous network. Key imaging properties are shown in Table 2. Figure 12 demonstrates the visual progression of this pipeline: the raw NIR data was effectively normalized using dual-stage CLAHE, which significantly enhanced local vessel visibility against the surrounding skin (Figure 12a,b). The subsequent application of the black-hat morphological filter and binarization successfully separated the dark vascular structures from background noise and surface hair artifacts, resulting in a clean, trackable vascular map (Figure 12c).

**Figure 13:**
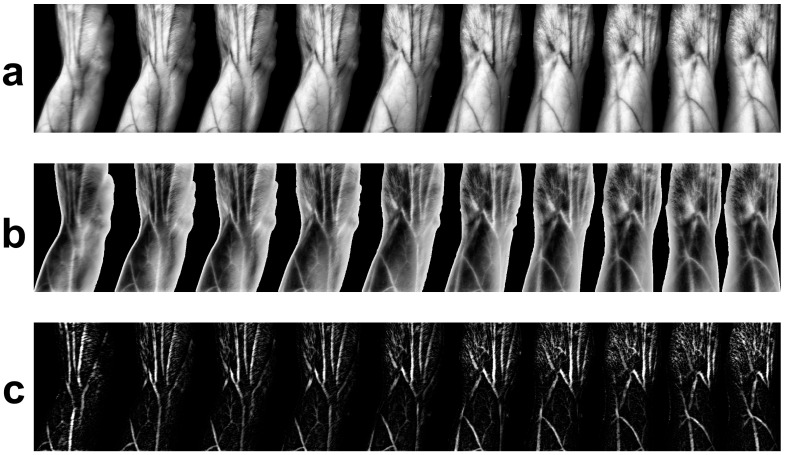
Image series of the left antecubital region illustrating the appearance of additional venous segments as a result of device rotation, 9.5 degrees per step is shown. (**a**) CLAHE unwrapped image sequence. (**b**) Inverse CLAHE unwrapped image sequence. (**c**) black-hat unwrapped image sequence. Example shown on a human subject.

**Figure 14:**
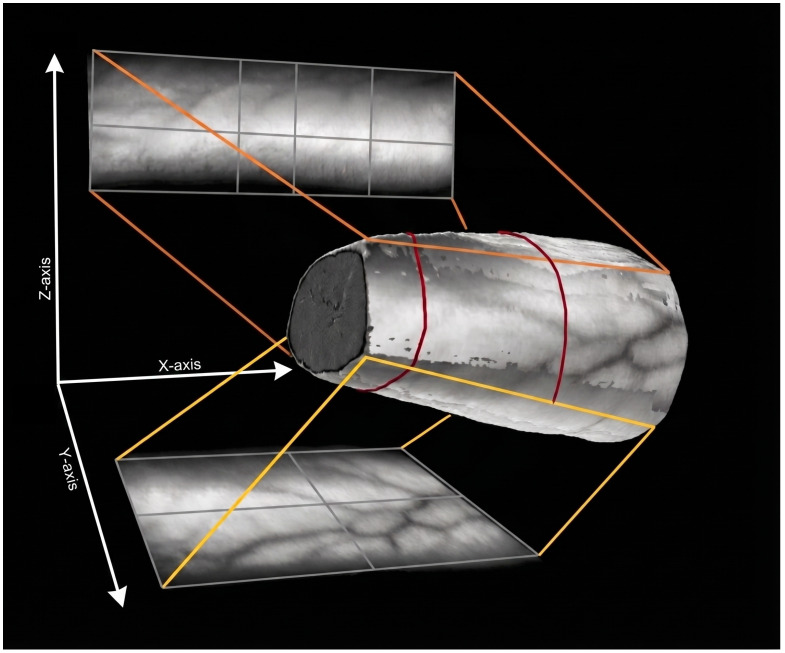
Qualitative 3D reconstruction from 2D panoramic projections, with texture mapping. The red rings illustrate topographical outlines on the reconstructed shell, presented for visual demonstration only. Example shown on a human subject.

By stitching the sequential frames, which are shown in Figure 13, we successfully obtained a panoramic projection that has been used as a texture map for the 3D reconstructed model. Figure 14 demonstrates a sample 3D reconstruction of the outer shell from the 2D projection outlines. The red rings illustrate localized circumference measurement locations on a single subject. The system-estimated values of 21.79 cm and 24.90 cm were comparable with the manual measurements of 21 cm and 24 cm, respectively. Furthermore, the system estimated the volume of the reconstructed limb segment to be 731.17 cm^3^. The manual ground truth measurement is prone to error; therefore, these results are included solely for demonstration purposes.

**Figure 15:**
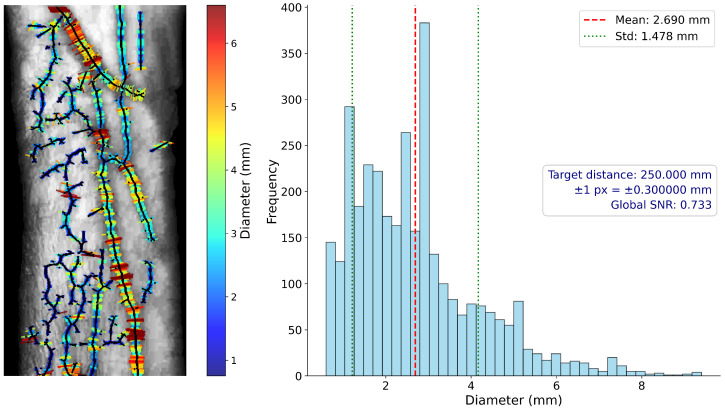
Vein diameter measurements with heatmap overlaid on the CLAHE enhanced image for easy localization. Example shown on a human subject.

Following the extraction and skeletonization of the venous network, cross-sectional diameters were automatically quantified. Figure 15 shows a representative mapping where the calculated vein diameters are rendered as a colormap overlaid on the CLAHE-enhanced image. The corresponding histogram shows the vein size distribution across the targeted region, demonstrating a mean superficial vein diameter of 2.690 mm (±1.478 mm).

## 4. Discussion

The primary objective of this study was to develop and evaluate a cost-effective, non-invasive panoramic NIR imaging system capable of automatically mapping and quantifying superficial vasculature. Our results demonstrate that the prototype successfully integrates rotational hardware, co-localized illumination, and a custom image processing pipeline to generate high-contrast 3D vascular maps and extract objective morphometric measurements. By characterizing the sensor array, we established that a 30 ms exposure time paired with a 3.5 analog gain optimally balanced the need to minimize motion blur during the panoramic sweep while maintaining a high SNR. Furthermore, as detailed in Appendix A, the integration of lateral mirrors enhanced photon collection. This hardware adjustment improved the detection of venous centerlines without necessitating longer exposure times or higher illumination intensities. The automated quantification pipeline yielded a mean superficial vein diameter of 2.69 mm on our human dataset, which closely aligns with anatomical expectations [11,29] and values reported by similar studies [17] for superficial veins situated 2–4.1 mm below the skin surface.

Currently, duplex ultrasound serves as the clinical gold standard for vascular diagnostics [1], and also for longitudinal tracking of certain vascular diseases [30]. However, its reliance on highly trained operators and its narrow cross-sectional field of view make wide-field, longitudinal assessment cumbersome and expensive. By contrast, the proposed NIR device provides an automated, operator-independent panoramic view, capable of imaging an approximately 35 cm long segment at once. While it does not replace the hemodynamic flow measurements provided by ultrasound, its ability to quickly document objective topological and morphological indices makes it highly suitable as a complementary first-line screening and monitoring tool. Commercial vein-finding devices (handheld 2D scanners) in clinical practice (such as AccuVein (AccuVein LLC, Cold Spring Harbor, NY, USA) and VeinViewer (Luminetx, Memphis, TN, USA)) use similar technologies [31] to visualize the vascular network. These tools are primarily utilized by nursing staff to facilitate venipuncture and cannula insertion by illuminating the skin surface from a single, fixed angle with the image of the identified vascular network region. Manual positioning of these handheld scanners inherently introduces operator-dependent subjectivity, making it difficult to standardize measurements or accurately document and assess the extent of superficial vascular diseases. Moreover, these devices do not allow saving the identified vascular network into traceable medical records; therefore, with these instruments longitudinal disease tracking is not currently feasible.

To validate the accuracy of the automated diameter quantification, we utilized synthetic phantoms generated as asymmetric fractal trees. These provided a mathematically predefined geometric ground truth containing small known-width branches in different orientations. These closely mimic the branching morphology and caliber variations of superficial venous networks. We rolled these printed patterns into 7 cm diameter tubes to replicate human limb segments during the imaging process. As demonstrated by the correlation analysis, the measured widths exhibited a strong positive correlation with the true values, tightly clustering along the ideal y=x line across all four distinct fractal morphologies. Furthermore, the Bland-Altman analysis revealed a consistent performance with a slight mean bias of 0.285 mm. This minor positive bias indicates a marginal overestimation of the vessel widths, which may be attributed to the blurring effect of the morphological filters or the discrete pixel-level nature of the spatial gradient calculations. Despite this small offset, the limits of agreement were acceptable, sub-millimeter values (−0.090 mm to 0.660 mm), demonstrating that the automated pipeline can reliably quantify vessel calibers with relatively small uncertainties. This geometric validation solidifies the system’s potential for tracking precise morphometric changes in clinical scenarios, but does not eliminate the need for further validation against clinically used ground truth techniques.

The clinical implications of such a system are broad, particularly for the longitudinal monitoring of conditions like venous insufficiency, superficial thrombosis, and localized inflammations. The co-registration of vascular mapping with 3D photogrammetric volume estimation further enhances our solution’s utility, allowing clinicians to obtain measurements required to track localized swelling or volumetric changes in tandem with vascular dilation. Furthermore, the method can be highly advantageous in geriatric care, where the limbs are inherently more fragile, and a superficial bump or injury triggers inflammatory and hemorrhagic reactions to a greater extent. Visualizing the exact scale and morphological impact of these localized reactions allows for a more precise, objective assessment of the trauma, ultimately facilitating the refinement of the therapeutic strategy.

Beyond the peripheral limbs, the fundamental physical principle of the reflective NIR tomographic arrangement allows for superficial vascular visualization on any anatomical region where veins are located close to the skin surface. Consequently, the technology holds significant potential for extension into other medical fields, such as urology or mammography. For instance, the non-invasive, contrast-enhanced mapping of superficial venous structures could serve as a valuable point-of-care tool in the diagnosis of superficial urological anomalies (e.g., varicoceles) or assist in precise pre-operative surgical planning, leveraging the exact same low-cost hardware and dual-stage CLAHE processing pipeline.

## 5. Conclusions

The proposed system addresses the limitations of narrow-field, operator-dependent modalities like duplex ultrasound by providing an accessible, wide-field approach to rapid and longitudinal superficial vascular monitoring. By integrating motorized rotational acquisition with a custom dual-stage CLAHE and structure-tensor-based processing pipeline, the prototype effectively mitigates the narrow field-of-view and operator-dependency limitations of conventional handheld devices. The system reliably isolated and quantified venous structures across a 35 cm field of view, achieving sub-millimeter geometric accuracy evaluated on synthetic fractal phantoms and yielding physiologically consistent in vivo measurements. This makes it a highly promising complementary tool for tracking morphological changes in superficial vessels.

## 6. Limitations and Future Research

The present study focused on the technical evaluation and demonstrating the feasibility of the experimental NIR prototype, with data collected from a limited cohort of 5 healthy volunteers. Consequently, future research will investigate the clinical utility and diagnostic sensitivity of these measurements in larger, longitudinal patient populations.

Although the mean superficial vein diameter quantified by the system aligns with established anatomical references, this study lacks a direct, patient-matched comparison against the clinical gold standard. Future clinical validation protocols will mandate synchronized duplex ultrasound measurements to rigorously confirm the absolute dimensional accuracy of the optical quantifications.

Several technical limitations are worth considering. First, the study cohort was small and only contained individuals with lighter skin tones. Given that melanin significantly influences NIR absorption and scattering at the current wavelength used [12,32], subjects with darker skin tones are expected to reflect fewer photons due to the higher epidermal melanin concentration. This attenuation could reduce the overall background intensity and decrease the optical contrast between the venous structures and the surrounding tissue, ultimately lowering the SNR. The performance of this system across diverse Fitzpatrick skin types remains to be validated. Future development will incorporate adaptive exposure compensation protocols to ensure efficacy across a more heterogeneous demographic.

During the measurements, the arms of the participants were not additionally supported to reduce movement; however, this has been accounted for by the processing software. Second, the proposed imaging device does not provide true cross-sectional volumetric data. Vessel diameters are estimated from 2D projections, which may be subject to errors resulting from the eventual non-circular morphology of actual vascular structures. Accordingly, this device is proposed as a complementary screening tool or a longitudinal tracking instrument for superficial vascular changes, rather than a replacement for established clinical standards such as ultrasonography or contrast-enhanced imaging in cases where true diameters are important. Ongoing work aims to refine algorithms to improve the accuracy of morphometric measurements.

Beyond clinical diagnostics, the high-contrast panoramic maps generated by the system, specifically the skeletonized vessel topologies, may hold significant potential for biometric authentication as well. By detecting deep-tissue NIR signatures, the device could serve as a dual-purpose platform for both medical screening and person identification.

## 7. Patents

Intellectual property protection application by B.J. (Application No. P2600282).

## Figures and Tables

**Table 1:** Baseline vital parameters of the healthy volunteers, during the experiment.

Gender	Age	Blood Pressure	SpO2
Male	27	127/75 mmHg	98%
Male	32	130/81 mmHg	99%
Female	33	128/73 mmHg	99%
Male	28	126/81 mmHg	98%
Female	55	132/78 mmHg	97%

**Table 2:** Key image-processing parameters across different human subjects.

Parameter Name	Subject 1	Subject 2	Subject 3	Subject 4	Subject 5
Average ROI Brightness	180.52	165.19	94.86	124.66	162.34
Ratio of Saturated Pixels	0.0%	0.0%	0.0%	0.0%	0.0%
Signal-to-Noise Ratio	6.24	5.97	19.452	7.688	6.525
Image Sharpness/Variance of Laplacian	5.41	5.75	2.6	3.36	4.16
RMS Contrast Gain	1.879×	2.434×	1.803×	1.884×	1.981×
Histogram Entropy	7 bit	6.674 bit	6.557 bit	6.69 bit	6.687 bit
Mean black-hat Response	27.50	33.9	20.6	29.48	26.57

**Figure A1:**
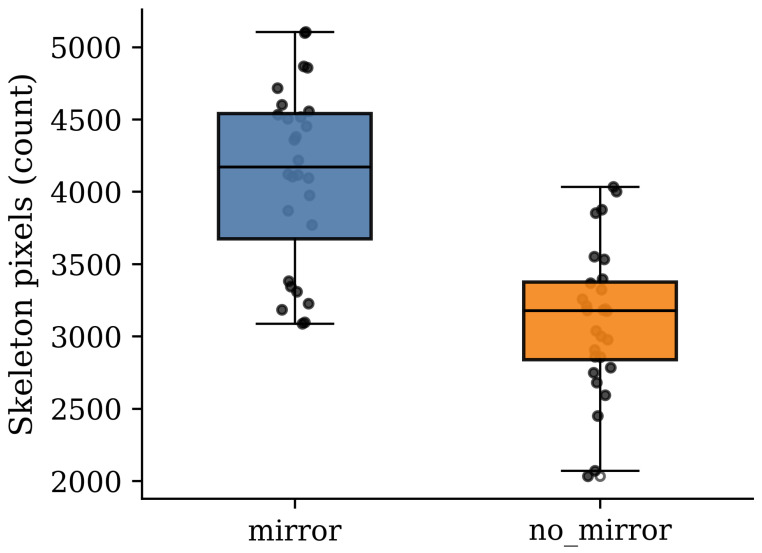
Identified vascular centerline (skeleton) pixels per frame across the two conditions.

**Figure A2:**
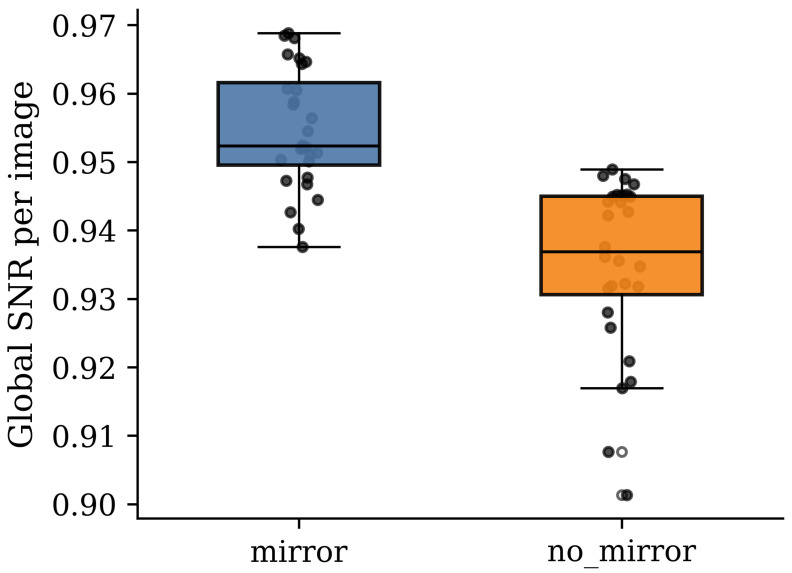
Comparison of Global Signal-to-Noise Ratio (SNR) per image with and without the use of lateral enhancement mirrors.

## Data Availability

The image processing scripts and non-sensitive data presented in this study may be provided upon reasonable request to the first author.

## Abbreviations

The following abbreviations are used in this manuscript:

NIR	Near-Infrared
CLAHE	Contrast Limited Adaptive Histogram Equalization
SpO2	Oxygen Saturation Percentage
LED	Light Emitting Diode
NoIR	Infrared Filterless Camera Module
FOV	Field Of View
SNR	Signal-to-Noise Ratio
ROI	Region Of Interest
RMS	Root Mean Square

## Appendix A

To evaluate the impact of the mirrors on the imaging performance, a comparative analysis was performed using rotational acquisition. It is important to note that this comparison was conducted on the same subject to isolate the purely optical effect of the lateral mirrors (i.e., enhanced photon collection) from the biological variance that would be present across multiple subjects. Two distinct image sequences were acquired with a minimal time delay on the same arm of a volunteer: one with the mirrors present and a control sequence in which the mirrors were masked with a non-reflective black material. For both conditions, equal frames were captured using a standardized 30 ms exposure time. Because the arm placement varied slightly between images and each frame captured a different rotational angle, direct frame-to-frame matching was not possible; therefore, the resulting image samples are considered robustly independent.

The Signal-to-Noise Ratio (SNR) was calculated to quantify the mirror-induced gain in image quality. After applying the image processing pipeline, a skeletonized map of the venous network was generated. The signal intensity ($\mu_{signal}$) was defined as the average pixel intensity across the width of the identified vascular structures. The background intensity ($\mu_{background}$) was determined by sampling a 5-pixel region on either side of the vessel; to avoid contamination from adjacent vascular structures or intersections, only values exceeding the local mean were included in the background calculation. The SNR was defined as:

(A1) $$SNR=\frac{\mu_{signal}}{\mu_{background}}\;$$

Initial evaluation of the processed sequences demonstrated a performance advantage when utilizing the mirrors. The imaging pipeline consistently identified more pixels as vein centerlines under the mirrored condition (averaging 4123.18 px per frame) compared to the masked control (averaging 3111.68 px per frame), as illustrated in Figure A1. Furthermore, the mirrored setup achieved a higher average SNR, yielding a mean SNR of 0.9547 (median: 0.9523) across n=28 images, compared to a mean of 0.9350 (median: 0.9369) across n=28 images without the mirrors. We observed the same effect when analyzing repeated acquisitions of the exact same static scene without camera movement, where the mirrored condition consistently produced a higher mean SNR (n=103 frames per condition). Based on these findings, we decided to keep the mirrors, although they are not essential.

Furthermore, the SNR distributions of the two independent conditions were compared statistically. For the dataset containing frames captured from multiple camera angles, statistical significance was evaluated on the mean SNR values of each frame using a non-parametric Mann-Whitney U test, as the data deviated from a normal distribution. As detailed in Table A1, the test indicated a significant difference between the two configurations, accompanied by an effect size of Rank-Biserial Correlation $r_{rb}=0.857$, 95% CI: [0.712, 0.964], statistically confirming the minimal advantage of the mirrored setup. The distribution of the global SNR across both imaging conditions is visualized in Figure A2.

**Table A1:** Statistical comparison of overall Signal-to-Noise Ratio (SNR) with and without lateral enhancement mirrors.

Statistical Parameter	Value
Sample Size (Mirror)	n=28
Sample Size (No Mirror)	n=28
Mean skeleton pixel count (Mirror)	4123.18
Mean skeleton pixel count (No Mirror)	3111.68
Mean SNR (Mirror)	0.9547
Mean SNR (No Mirror)	0.9350
Test Statistic (*U*)	728.00
*p*-value	$3.846\times 10^{-8}$
Rank-Biserial Correlation ($r_{rb}$)	0.857
95% Bootstrap CI	[0.712, 0.964]

## References

[1] Garcia R., Labropoulos N. (2018). Duplex Ultrasound for the Diagnosis of Acute and Chronic Venous Diseases. Surg. Clin. N. Am..

[2] Bing S., Huang M.H., Cao H., Chiao J.C. (2026). Feasibility Study of Noninvasive Subcutaneous Imaging for Vein Localization. Electronics.

[3] Goyen M., Ruehm S.G., Debatin J.F. (2000). MR-angiography: The role of contrast agents. Eur. J. Radiol..

[4] Altay A., Gumus A. (2024). Real-time superficial vein imaging system for observing abnormalities on vascular structures. Multimed. Tools Appl..

[5] Miyake R.K., Zeman H.D., Duarte F.H., Kikuchi R., Ramacciotti E., Lovhoiden G., Vrancken C. (2006). Vein Imaging: A New Method of Near Infrared Imaging, Where a Processed Image Is Projected onto the Skin for the Enhancement of Vein Treatment. Dermatol. Surg..

[6] Kauba C., Prommegger B., Uhl A. (2019). Combined Fully Contactless Finger and Hand Vein Capturing Device with a Corresponding Dataset. Sensors.

[7] Kauba C., Drahanský M., Nováková M., Uhl A., Rydlo Š. (2022). Three-Dimensional Finger Vein Recognition: A Novel Mirror-Based Imaging Device. J. Imaging.

[8] Jacques S.L. (2013). Optical properties of biological tissues: A review. Phys. Med. Biol..

[9] Tseng S.H., Bargo P., Durkin A., Kollias N. (2009). Chromophore concentrations, absorption and scattering properties of human skin in-vivo. Opt. Express.

[10] Delpy D.T., Cope M. (1997). Quantification in tissue near–infrared spectroscopy. Philos. Trans. R. Soc. Lond. B Biol. Sci..

[11] Zharov V.P., Ferguson S., Eidt J.F., Howard P.C., Fink L.M., Waner M. (2004). Infrared imaging of subcutaneous veins. Lasers Surg. Med..

[12] Roy S., Wu J., Cao J., Disu J., Bharadwaj S., Meinert-Spyker E., Grover P., Kainerstorfer J.M., Wood S. (2024). Exploring the impact and influence of melanin on frequency-domain near-infrared spectroscopy measurements. JBO.

[13] Zhang S.X., Schmidt H.M. (1993). Clinical anatomy of the subcutaneous veins in the dorsum of the hand. Ann. Anat.-Anat. Anz..

[14] Yang Z., Shi M., Gharbi Y., Qi Q., Shen H., Tao G., Xu W., Lyu W., Ji A. (2024). A Near-Infrared Imaging System for Robotic Venous Blood Collection. Sensors.

[15] Marshall S.K., Cheewakul J., Ina N., Rojchanaumpawan T., Booranawong A. (2026). The Development and Experimental Evaluation of a Non-Invasive Vein Visualization System Using a Near-Infrared Light Source and a Web Camera to Assist Medical Personnel in Radiology Contrast Administration and Venous Access. Appl. Sci..

[16] Ndoumbe J., Akoa B.E., Talotsing G.P., Kounga F.F., Kaissassou S., Njo B.C. (2024). Automatic Detection and Characterization of Human Veins Using Infra-Red Image Processing. J. Comput. Commun..

[17] Silva H., Rezendes C. (2025). Texture Analysis of Near-Infrared Vein Images During Reactive Hyperemia in Healthy Subjects. Appl. Sci..

[18] Prommegger B., Kauba C., Uhl A. (2018). Multi-Perspective Finger-Vein Biometrics. Proceedings of the 2018 IEEE 9th International Conference on Biometrics Theory, Applications and Systems (BTAS).

[19] Ishaq M.H., Hashim Ali R., Koutaly R., Khan T.A., Ahmad I. (2025). Enhanced Biometric Security Through Infrared Vein Pattern Recognition. Proceedings of the 2025 International Conference on Innovation in Artificial Intelligence and Internet of Things (AIIT).

[20] Kang W., Liu H., Luo W., Deng F. (2019). Study of a Full-View 3D Finger Vein Verification Technique. IEEE Trans. Inf. Forensics Secur..

[21] Rotunno G., Salvi M., Deinsberger J., Krainz L., Weber B., Sinz C., Kittler H., Schmetterer L., Drexler W., Liu M. (2025). DERMA-OCTA: A Comprehensive Dataset and Preprocessing Pipeline for Dermatological OCTA Vessel Segmentation. Sci. Data.

[22] Huynh N.T., Zhang E., Francies O., Kuklis F., Allen T., Zhu J., Abeyakoon O., Lucka F., Betcke M., Jaros J. (2025). A fast all-optical 3D photoacoustic scanner for clinical vascular imaging. Nat. Biomed. Eng..

[23] Ndu H., Sheikh-Akbari A., Deng J., Mporas I. (2024). HyperVein: A Hyperspectral Image Dataset for Human Vein Detection. Sensors.

[24] Mahmoud A., El-Sharkawy Y.H. (2023). Quantitative phase analysis and hyperspectral imaging for the automatic identification of veins and blood perfusion maps. Photodiagnosis Photodyn. Ther..

[25] Wołk K., Wołk A. (2025). Hyperspectral Imaging System Applications in Healthcare. Electronics.

[26] Pizer S.M., Amburn E.P., Austin J.D., Cromartie R., Geselowitz A., Greer T., ter Haar Romeny B., Zimmerman J.B., Zuiderveld K. (1987). Adaptive histogram equalization and its variations. Comput. Vis. Graph. Image Process..

[27] Said H., Mohamed S., Shalash O., Khatab E., Aman O., Shaaban R., Hesham M. (2024). Forearm Intravenous Detection and Localization for Autonomous Vein Injection Using Contrast-Limited Adaptive Histogram Equalization Algorithm. Appl. Sci..

[28] Otsu N. (1979). A Threshold Selection Method from Gray-Level Histograms. IEEE Trans. Syst. Man Cybern..

[29] Ganesh S. (2007). Depth and Size Limits for the Visibility of Veins Using the VeinViewer Imaging System. Master’s Thesis.

[30] Rathbun S.W., Aston C.E., Whitsett T.L. (2012). A randomized trial of dalteparin compared with ibuprofen for the treatment of superficial thrombophlebitis. J. Thromb. Haemost..

[31] Cuper N.J., Verdaasdonk R.M., de Roode R., de Vooght K.M.K., Viergever M.A., Kalkman C.J., de Graaff J.C. (2011). Visualizing Veins With Near-Infrared Light to Facilitate Blood Withdrawal in Children. Clin. Pediatr..

[32] Pachyn E., Aumiller M., Freymüller C., Linek M., Volgger V., Buchner A., Rühm A., Sroka R. (2024). Investigation on the influence of the skin tone on hyperspectral imaging for free flap surgery. Sci. Rep..

